# Transmission of Carbapenem-Resistant *Enterobacterales* in an Overcrowded Emergency Department: Controlling the Spread to the Hospital

**DOI:** 10.1093/cid/ciad263

**Published:** 2023-07-05

**Authors:** Matias C Salomão, Maristela P Freire, Carolina S Lázari, Ana P Cury, Flávia Rossi, Aluisio A C Segurado, Silvia F Costa, Anna S Levin, Ícaro Boszczowski, Carbapenem Resistant Enterobacterales in Emergency Department (CRE-ED) Task Force, Raphael B R Tolentino, Laina Bubach, Bianca L Almeida, Lia M Barreira, Priscilla C Saihg, Roberta V P Yokogawa, Ana Rubia Guedes, Thais Guimarães

**Affiliations:** Department of Infectious Diseases, Faculdade de Medicina; Infection Control Department; Department of Infectious Diseases, Faculdade de Medicina; Infection Control Department; Divisão de Patologia Clínica, Departamento de Patologia, Laboratório de Investigação Medica (LIM03); Departamento de Patologia, Central Microbiology Laboratory, LIM03 Division; Departamento de Patologia, Central Microbiology Laboratory, LIM03 Division; Department of Infectious Diseases, Faculdade de Medicina; Department of Infectious Diseases, Hospital das Clínicas, Universidade de São Paulo, Brazil; Department of Infectious Diseases, Faculdade de Medicina; Infection Control Department; Department of Infectious Diseases, Hospital das Clínicas, Universidade de São Paulo, Brazil; Department of Infectious Diseases, Faculdade de Medicina; Infection Control Department; Department of Infectious Diseases, Hospital das Clínicas, Universidade de São Paulo, Brazil; Department of Infectious Diseases, Faculdade de Medicina; Infection Control Department

**Keywords:** carbapenem-resistant *Enterobacterales*, screening, emergency department, multidrug resistance, PCR

## Abstract

**Background:**

Overcrowded emergency departments (EDs) may increase the risk of carbapenem-resistant *Enterobacterales* (CRE) transmission.

**Methods:**

We conducted a quasi-experimental study divided into 2 phases (baseline and intervention) to investigate the impact of an intervention on the acquisition rate and identify risk factors for CRE colonization in an ED of a tertiary academic hospital in Brazil. In both phases, we did universal screening with rapid molecular test (*bla*_KPC_, *bla*_NDM_, *bla*_OXA48_, *bla*_OXA23_, and *bla*_IMP_) and culture. At baseline, both screening test results were not reported, and patients were put under contact precautions (CP) based on previous colonization or infection by multidrug-resistant organisms. During the intervention, all patients hospitalized in the ED were placed in empiric CP and the result of CRE screening was reported; if negative, patients were released from CP. Patients were rescreened if they stayed >7 days in the ED or were transferred to an intensive care unit.

**Results:**

A total of 845 patients were included: 342 in baseline and 503 in intervention. Colonization at admission was 3.4% by culture and molecular test. Acquisition rates during ED stay dropped from 4.6% (11/241) to 1% (5/416) during intervention (*P* = .06). The aggregated antimicrobial use in the ED decreased from phase 1 to phase 2 (804 defined daily doses [DDD]/1000 patients to 394 DDD/1000 patients, respectively). Length of stay >2 days in the ED was a risk factor for CRE acquisition (adjusted odds ratio, 4.58 [95% confidence interval, 1.44–14.58]; *P* = .01).

**Conclusions:**

Early empiric CP and rapid identification of CRE-colonized patients reduce cross-transmission in ED. Nevertheless, staying >2 days in ED compromised efforts.

## Teaser

This quasi-experimental study shows that early contact precautions and rapid identification of patients colonized with carbapenem-resistant *Enterobacterales* was associated with a nonsignificant reduction of cross-transmission in emergency departments.

*Enterobacterales* are gram-negative bacteria and important causes of healthcare-associated infections. The emergence of enzymes capable of hydrolyzing a wide range of antimicrobials has led to a general increase in antimicrobial resistance [1–5].

An essential factor for disseminating these enzymes among *Enterobacterales* is that these carbapenemase genes are located in plasmids, which allows their easy interspecies transmission. The intestinal colonization of carbapenem-resistant *Enterobacterales* (CRE) can act as an important reservoir and potential disseminator of in-hospital resistance [6–9].

Tests focused on detecting carbapenemases can identify carbapenemase-harboring organisms without the need for cultures and antimicrobial susceptibility tests, reducing turnaround time. Nevertheless, their high cost is still a significant obstacle to their application [10].

In our hospital, the average CRE colonization rate on admission to an intensive care unit (ICU) is 5%–7% (unpublished data). Notably, about 30% of patients admitted to ICUs come from the emergency department (ED), which could be a source of patients colonized by CRE. In 2017, our group identified that 6.8% of patients admitted to the ED were colonized with CRE. Secondary colonization occurred in 18% of patients, indicating that the ED is a potential source of acquisition and spread of resistance to the entire hospital [11]. This may be due to local conditions such as overcrowded EDs with relatively long length of stay (LOS) (median, 4 days). In 2019, 40% of patients had their health needs resolved during their stay in the ED without occupying a bed in a hospital ward or ICU. This situation is common in many public EDs in Brazil and may be a huge undetected hot spot for multidrug-resistant organism (MDRO) transmission within facilities. Interventions in this setting may have an impact on the whole chain of CRE transmission in the hospital. We performed a study with the following objectives: (1) to evaluate the impact of an intervention on CRE acquisition rate in patients during their ED stay and (2) to identify risk factors for CRE colonization during ED stay.

## METHODS

This study was conducted in the ED of an 800-bed university tertiary hospital affiliated with the University of São Paulo, in Brazil. The ED is overcrowded, often having twice as many patients hospitalized as its maximum capacity, and it is not uncommon for patients to be hospitalized in the ED for >11 days [11]. Our hospital was assigned to be dedicated to coronavirus disease 2019 (COVID-19) from 1 April to 31 August 2020. After August 2020, the hospital was gradually reopened for other admissions.

This was a quasi-experimental study divided into 2 phases. Phase 1 (baseline period) was performed from 3 to 28 February 2020 (before the first case of COVID-19 in our facility), and phase 2 (intervention period) from 14 September to 1 October 2020.

Since 2013, as a hospital routine, we place patients under preemptive contact precautions (CP) and collect rectal swab cultures for CRE screening for all patients admitted to the ICU. Patients who are CRE positive are kept under CP, and the others are released. Once a week, all patients in the ICU are rescreened for CRE with rectal swab culture. Patients under CP are kept in individual rooms or in cohorts with other CRE-colonized patients. These screening measures are not routinely applied in other areas such as the ED or regular wards.

Throughout the study, we did universal screening for CRE by phenotypic (culture with carbapenem susceptibility testing) and molecular methods using real-time polymerase chain reaction (rt-PCR) for *bla*_KPC_, *bla*_NDM_, *bla*_IMP_, *bla*_VIM_, and *bla*_OXA48_ genes (Xpert Carba-R, Cepheid, Sunnyvale, California). During both phases, all patients admitted to the ED had 2 rectal swabs collected: 1 for culture and another for rt-PCR. Patients whose screening for CRE on admission was negative were considered to be noncolonized on admission. Patients who remained for at least 7 days in the ED had another pair of swabs collected weekly until transfer to another unit, another acute care hospital, or discharge home. Patients transferred to an ICU were also screened for CRE upon ICU admission with a rectal swab collected for culture within the first 24 hours of ICU admission, even if they were <7 days in the ED.

Patients whose initial ED rectal swab was negative and whose subsequent swab collected in the ED or <24 hours after the transfer to an ICU was positive for CRE (rt-PCR or culture) were considered to have acquired CRE in the ED, independent of whether they were under CP or not.

Phase 1 consisted of a baseline period to determine the prevalence and incidence of CRE-colonized patients admitted to the ED. No intervention was done in phase 1. As part of standard ED care, patients who stay >24 hours are admitted to the ED and placed on stretchers and beds distributed close to each other in halls and corridors while waiting to be transferred to a definitive unit. The standard of practice in ED is to only place under CP patients who were known to be colonized by an MDRO. The results of the CRE screening were not available in the hospital information system and the attending staff did not know the results of the CRE screening.

Phase 2 was the intervention period consisting of universal empirical contact and respiratory precautions upon entrance for patients admitted to the ED. While awaiting PCR for severe acute respiratory syndrome coronavirus 2 (SARS-CoV-2), patients were placed in a dedicated area. Rectal swabs for CRE screening were collected from patients within the cohort whose admission was indicated in the ED. This was performed within the first 24 hours of hospitalization in the ED. Patients with positive PCR for carbapenemase genes, regardless of their SARS-CoV-2 result, remained under CP until ED discharge. In the intervention phase, results were available in the hospital information system.

The cleaning and disinfection procedures in both phases were similar. For stretchers and beds, the cleaning was made using a 70% alcohol solution. In phase 2, if the beds were in a separate room, a terminal cleaning was also performed, using 70% alcohol, ammonium quaternary disinfectant, and hypochlorite, according to the surface that was cleansed.

Clinical, demographic, and epidemiologic data were collected from all patients on admission to the ED during phases 1 and 2 to analyze risk factors for CRE colonization.

Infection control personnel monitored hand hygiene (HH) compliance during both phases of the study using the World Health Organization “5 Moments” audit tool [12].

CRE colonization pressure and carbapenem-resistant *Klebsiella pneumoniae* infection rates were monitored continuously in the 2 phases in the ICU. *Klebsiella pneumoniae* is the most prevalent CRE in our hospital, representing up to 90% of the CRE isolates.

Antimicrobial use was monitored in both phases. Data are presented in aggregated defined daily doses (DDD) per 1000 patients for all antibiotics.

## Microbiology

For colonization culture, the rectal swabs were enriched in liquid media (thioglycolate 8 mL) overnight; the broth was then subcultured to a MacConkey agar plate with ertapenem, imipenem, and meropenem discs. Following overnight incubation, resistant isolates were directly identified using matrix-assisted laser desorption/ionization time-of-flight mass spectrometry (bioMérieux, Marcy-l’Etoile, France). We considered all *Enterobacterales* resistant to any of those drugs as CRE. The average turnaround time for this test is 48–72 hours.

### Molecular Biology

Resistance genes were detected directly from the rectal swabs. The samples were evaluated by rt-PCR for *bla*_KPC_, *bla*_NDM_, *bla*_IMP_, *bla*_VIM_, and *bla*_OXA-48_ with Cepheid Xpert Carba-R according to the manufacturer's recommendations. The molecular biology laboratory runs the tests twice daily, 7 days a week. Thus, the time lag between obtaining the sample and publishing the results on the hospital information system varied from 1 hour to 12 hours.

### Sample Size

We estimated a sample size of 560 patients on a 1.5 enrollment ratio, for an α of .05, power of 80%, considering a decrease in CRE acquisition rate from 5% to 1%.

### Data Analysis

Admission and follow-up (secondary) CRE colonization rates in the ED were compared between phases 1 and 2, and stratified by LOS in the ED.

Noncolonized patients on admission who became colonized were compared to noncolonized patients who remained noncolonized during their ED stay. CRE-colonized patients on admission were compared to noncolonized patients. Covariates associated with CRE colonization on bivariate analysis were included in the logistic regression model to evaluate independent risk factors for CRE colonization. A 2-tailed *P* value <.05 was considered statistically significant. Statistical analyses were performed using EPI Info version 7.2 (Centers for Disease Control and Prevention, Atlanta, Georgia).

This study was approved by the Hospital das Clínicas Ethics Committee (Certificate of Presentation for Ethical Consideration: 50769521.0.0000.0068).

## RESULTS

Eight hundred forty-five patients were included in this study: 342 in phase 1 and 503 in phase 2. Thirteen patients (2%) refused to participate in the study. Fifty percent (171 and 255 patients in phases 1 and 2, respectively) were female, and the median age was 58 years in both phases (Table 1).

**Table 1.** Clinical and Demographic Characteristics of Patients Admitted to the Emergency Department, Hospital das Clínicas, University of São Paulo, Brazil, 2020

Variable	All Patients (N = 845)	Phase 1 (Baseline) (n = 342)	Phase 2 (Intervention) (n = 503)	*P* Value
Sex (female)	419 (50)	171 (50)	255 (51)	.84
Age, y, median (range)	58 (3–97)	58 (3–96)	58 (3–97)	.97
Comorbidities	493 (58)	182 (53)	311 (62)	.01
Hypertension	273 (32)	94 (27)	179 (31)	.01
Diabetes	206 (12)	40 (12)	65 (13)	.84
Cardiovascular disease	58 (7)	15 (4)	43 (8)	.02
Lung disease	18 (2)	4 (1)	14 (3)	.11
Liver disease	31 (4)	12 (3)	19 (4)	.84
Renal disease	51 (6)	16 (5)	35 (7)	.17
Stroke	83 (10)	37 (11)	46 (9)	.42
Cancer	47 (6)	15 (4)	32 (6)	.22
Solid organ transplantation	23 (3)	10 (9)	13 (3)	.77
Onco-hematologic disease	10 (1)	2 (0.6)	8 (2)	.22
Autoimmune disease	36 (4)	13 (4)	23 (5)	.59
Other comorbidities	127 (15)	51 (15)	76 (15)	.94
Exposed to healthcare in the last year	209 (25)	63 (18)	146 (29)	<.001
Surgery in the last 30 d	35 (4)	16 (5)	19 (4)	.52
Antibiotic use in the last 30 d	166 (20)	51 (15)	92 (18)	.20
Category of ED admission				
Surgical emergency	104 (12)	43 (13)	61 (12)	.85
Clinical emergency	650 (77)	288 (84)	362 (72)	<.001
Trauma	55 (6)	19 (6)	36 (7)	.40
Mortality and infection				
In-hospital mortality	89 (11)	33 (10)	56 (11)	.49
HAI during hospitalization	6 (1)	2 (1)	4 (1)	.72

Data are presented as No. (%) unless otherwise indicated.

Abbreviations: ED, emergency department; HAI, healthcare-associated infection.

CRE colonization at ED admission was similar between phase 1 and phase 2 (4% vs 3%, *P* = .51) (Supplementary Table 1). One patient in phase 1 and 2 patients in phase 2 were known to have been colonized by CRE in the 6 months that preceded their hospitalization. One of these patients in phase 2 was colonized on the ED admission.

Two hundred forty-one (70%) patients had a second test collected for CRE screening in phase 1, showing that 5% (11/241) of patients had become colonized during their ED stay. In phase 2, 415 patients (83%) had another test collected for CRE after ED admission, and 1% (5/416) became colonized during their ED stay (Table 2). Thirty-two of the 656 (5%) patients who were rescreened had the second test collected in the ED. The average LOS in the ED was 5.44 days and 1.28 days in phases 1 and 2, respectively. In the same period, the average LOS in the ICUs remained stable (phase 1, 9.42 days; phase 2, 8.42 days). The mean time between ED admission and a second test was 1.43 (median, 1 [range, 0–21]) days and 1.14 (median, 1 [range, 0–13]) days for phases 1 and 2, respectively. One hundred (29%) patients in phase 1 and 88 (17%) patients in phase 2 were discharged from the hospital without a second CRE screening test. In the bivariate analysis, the intervention had a protective effect on the risk of becoming colonized by CRE in the ED (odds ratio [OR], 0.31 [95% confidence interval {CI}: .10–.94]; *P* = .04). However, after adjustments with confounding variables in the multivariate model, it did not sustain its effect (adjusted OR [aOR], 0.34 [95% CI: .11–1.07]; *P* = .06) (Table 3).

**Table 2.** Rates of Carbapenem-Resistant *Enterobacterales* Colonization at Admission and Acquisition During the Emergency Department Stay, Hospital das Clinicas, University of São Paulo, Brazil, 2020

CRE Colonization	All Patients (N = 845)	Phase 1 (Baseline) (n = 342)	Phase 2 (Intervention) (n = 503)	OR (95% CI)	*P* Value
At admission to the ED	28 (3)	13 (4)	15 (3)	0.78 (.37–1.66)	.65
Acquisition during ED stay	16/657 (2)	11/241 (5)	5/416 (1)	0.25 (.09–.74)	.01
Acquisition during ED stay ≤2 d	6/593 (1)	4/205 (1)	2/388 (1)	0.26 (.05–1.43)	.12
Acquisition during ED stay >2 d	10/112 (9)	7/85 (8)	3/27 (11)	1.39 (.33–5.80)	.65

Data are presented as No. (%) unless otherwise indicated. Numerators vary according to the number of patients who were rescreened in each period.

Abbreviations: CI, confidence interval; CRE, carbapenem-resistant *Enterobacterales*; ED, emergency department; OR, odds ratio comparing phases 1 and 2.

**Table 3.** Risk Factors for Carbapenem-Resistant *Enterobacterales* Colonization During Emergency Department (ED) Stay in a Cohort of Patients Admitted to the ED, Hospital das Clínicas, University of São Paulo, Brazil, 2022 (n = 657)

Variable	Not CRE Colonized During ED Stay (n = 641)	CRE Colonized During ED Stay (n = 16)	Bivariate Analysis			Multivariate Analysis		
			OR	(95% CI)	*P* Value	aOR	(95% CI)	*P* Value
Sex (female)	324 (51)	3 (19)	3.74	(1.03–13.56)	.06	…	…	
Age (>65 y)	243 (38)	7 (44)	1.23	(.42–3.58)	.71	…	…	
Previous comorbidities	390 (61)	10 (63)	1.16	(.38–3.50)	.79	…	…	
Hypertension	208 (32)	5 (31)	1.16	(.38–3.49)	.78	…	…	
Heart disease	50 (8)	0 (0)	0	…	.61	…	…	
Lung disease	15 (2)	1 (6)	3.21	(.39–26.15)	.29	…	…	
Liver disease	50 (8)	3 (19)	7.33	(1.91–28.06)	.02	7.69	(1.90–31.14)	<.01
Kidney disease	43 (7)	3 (19)	2.32	(.50–10.69)	.25	…	…	
Stroke	66 (10)	0 (0)	0	…	.38	…	…	
Cancer	39 (6)	0 (0)	2.57	(.56–11.90)	.22	…	…	
Solid organ transplant	21 (3)	0 (0)	0	…	1	…	…	
Hematologic malignancy	10 (2)	0 (0)	0	…	1	…	…	
Autoimmune disease	28 (4)	0 (0)	0	…	1	…	…	
Other comorbidities	96 (15)	3 (19)	1.55	(.42–5.65)	.46	…	…	
Exposure to healthcare in last year	165 (26)	4 (25)	0.79	(.22–2.86)	1	…	…	
Use of antibiotics in last 90 d	162 (25)	4 (25)	1.09	(.30–3.98)		…	…	
Reason for ED hospitalization								
Surgical emergency	77 (12)	3 (19)	2.00	(.55–7.32)	.29	…	…	
Clinical emergency	380 (59)	12 (75)	1.72	(.54–5.53)	.36	…	…	
Neurological emergency	67 (10)	0 (0)	0	…	.38	…	…	
Pulmonary emergency	29 (5)	1 (6)	1.62	(.21–12.83)	.64	…	…	
Trauma	49 (8)	0 (0)	0	…	.61	…	…	
Acute myocardial infarction	8 (1)	0 (0)	0	…	1	…	…	
Study phase								
Baseline	230 (36)	11 (69)		…		…	…	
Intervention	411 (64)	5 (31)	0.31	(.10–.94)	.04	0.34	(.11–1.07)	.06
ED hospitalization								
>2 d	6/593 (1)	10/112 (9)	5.69	(1.84–17.56)	<.01	4.58	(1.44–14.58)	.01

Data are presented as No. (%) unless otherwise indicated.

Abbreviations: aOR, adjusted odds ratio; CI, confidence interval; CRE, carbapenem-resistant *Enterobacterales*; ED, emergency department; OR, odds ratio.

CRE colonization increased with time spent in the ED. Patients who spent >2 days in the ED had an aOR of 4.58 (95% CI: 1.44–14.58; *P* = .01) of acquiring CRE during their stay when compared to patients who stayed up to 2 days in the ED. Patients who had a chronic liver disease were also at higher risk of becoming colonized by CRE during their ED stay (aOR, 7.69 [95% CI: 1.90–31.14]; *P* < .01) (Table 3).

The only independent risk factor for being colonized by CRE on ED admission was having a pulmonary emergency as cause of admission (aOR, 3.22 [95% CI: 1.06–9.81]; *P* = .04) (Table 4).

We observed 469 HH opportunities in both periods. Compliance was 70% (124/177) in phase 1 and 37% (110/292) in phase 2.

The aggregated antimicrobial use in the ED decreased from phase 1 to phase 2 (804.04 DDD/1000 patients to 394.29 DDD/1000 patients, respectively).

In the first trimester of 2020, the carbapenem-resistant *K. pneumoniae* infection incidence in ICUs was 1.2 infections per 1000 patient-days and in the third trimester, it dropped to 0.9 infections per 1000 patient-days. Similarly, the CRE colonization pressure in ICUs also decreased from 12% to 7% (Supplementary Figures 1 and 2).

## DISCUSSION

We assessed whether universal empiric CP and CRE screening could significantly reduce cross-transmission in an overcrowded ED with long LOS. During the intervention period, CRE acquisition dropped by 66%; however, it was not statistically significant. Staying >2 days in the ED was a risk factor for becoming colonized by CRE.

There was not a significant difference in the overall colonization rate on ED admission between the baseline and intervention periods. The only independent risk factor associated with CRE colonization on admission to ED across phases was having a pulmonary emergency as cause of admission to the ED. Although it suggested that a comorbidity would also be associated, this association was not found in the bivariate analysis.

All ED staff were periodically trained on HH, personal protective equipment use, and adherence to CP on both phases. However, adherence to HH dropped significantly from phase 1 to phase 2, which may have contributed to reduce the impact of the intervention on the acquisition of CRE in the ED. This poor performance in HH during phase 2 may be associated with the impact of the COVID-19 pandemic on the routine of care. This phenomenon was also described by other authors, with low and worse HH compliance during the COVID-19 pandemic [13, 14]. Our hospital had an overcrowded ED dedicated to COVID-19 patients from April to August 2020. From March 2020 to July 2021, the staff was reinforced in the ED due to the COVID-19 response. From phase 1 to phase 2, the nurse to patient ratio stayed the same (1 nurse/10 patients), and the nurse assistant (NA) to patient ratio improved from 1 NA per 5 patients to 1 NA per 4 patients. In September 2020, the hospital reopened for new admissions for other medical and surgical specialties. Nonetheless, at the ED, all patients were admitted in contact and respiratory precautions until their PCR for SARS-CoV-2 was negative. Regardless of the test result, all colonized patients with CRE remained under CP. We hypothesize that given the high number of patients under CP, the healthcare workers adhered less to HH. This is a phenomenon previously observed by different authors [15].

Our study focused on the ED because we previously detected this unit as an important reservoir of CRE in the hospital. It played an important role in the dissemination of these pathogens to other areas of the hospital, including ICUs [16]. We hypothesized that if patients were put under empiric CP, promptly identified by rapid molecular tests and segregated in a dedicated cohort, the acquisition rate while in the ED could be contained. In this study, we observed a non–statistically significant decrease in the incidence of the acquisition rate of CRE after the intervention. Furthermore, if the patient stayed >2 days in the ED, the effect of the intervention seemed to decrease. The decrease in CRE acquisition was almost significant with the intervention and we hypothesize that we may not have had enough time to confirm the intervention’s impact in this analysis. It is possible that by increasing the number of patients tested, we could have observed a difference. Another explanation is that regardless of the rapid CRE colonization status identification by molecular tests, patients require allocation to a definitive hospital bed (ICU or non-ICU), as the ED’s infrastructure is not adequate enough to implement infection prevention and control (IPC) strategies to stop the spread of CRE. Moreover, although out of the scope of this study, a long stay in the ED may pose other adverse event risks.

Overcrowding leads to patients staying in stretchers very close to each other. This scenario added to the universal CP and staff overwhelmed by the workload and may pose an increased risk of failure to adhere to IPC strategies; the risk increases with time, as we observed in the low HH compliance. Average LOS in the ED decreased from phase 1 to phase 2. Even so, we found that staying >2 days in the ED is an independent risk factor for becoming colonized by CRE. This result is similar to what was found in a previous study by our group, in which staying >2 days in the ED was a risk factor for arriving at the ICU colonized with CRE [16]. We conclude, based on our results, that it is necessary to remove patients from the ED within 2 days at most. Overcrowded EDs are a public health issue in many countries across the globe, and this phenomenon has different reasons related to the local healthcare systems and intrafacility operation management. The scenario found in our ED is common to many other EDs in Brazil and in other developing countries. Prolonged hospitalization and high mortality in patients hospitalized in the ED are reported in other hospitals in Brazil [17–19]. Despite being a single-center study, our findings may be applicable to other developing countries.

Our study has several limitations. We planned an intervention that included a dedicated cohort of patients on CP after prompt identification by rapid molecular tests on admission to the ED. The original design was affected by the crisis triggered by the COVID-19 pandemic. Although during the intervention phase, all patients were admitted on CP because of COVID-19 transmission prevention and remained after CRE identification regardless of a negative SARS-CoV-2 molecular test, the adherence to HH was low and there may have been an inadequate use of CP. We did not audit CP compliance during the study. We did not audit stretcher, bed, and room cleaning and disinfection procedures in either phase. As most patients in the ED remained under CP, we believe that healthcare workers may have neglected the need to change gowns and gloves between patients due to the work overload. This was a single-center study, and the results found may not be generalizable to other centers. Our hospital was one of the referral hospitals of the state of São Paulo for COVID-19 care during 6 consecutive months. We had 250 ICU beds and another 250 ward beds dedicated to COVID-19 admissions at the peak of the first COVID-19 wave in Brazil. Thus, the patient case mix has completely changed, and the analysis of this period is not generalizable to other scenarios, even at our facility. Even so, we observed a downward trend in the CRE acquisition rate during phase 2. We hypothesize that early identification and cohorting of colonized patients contributed to this trend. Moreover, in a scenario of universal CP and high workload due to COVID-19 patients in our ICUs, the colonization pressure did not increase during the intervention phase (Supplementary Figure 2).

Interventions in the ED to control CRE transmission to the hospital are necessary, especially in overcrowded EDs. CRE universal screening, with rectal swab culture or rapid molecular tests, is an interesting tool for the prompt identification of patients colonized by CRE and, if possible, the allocation of patients into cohorts, reducing cross-transmission. However, if this resource is not coupled with a comprehensive management plan for informing results to the healthcare workers, patient allocation in definitive beds across the facility, and robust good practices of IPC, the goal may not be achieved. Staying >2 days in the ED is a risk factor for becoming colonized by CRE and this probably is a good target for a new intervention. The IPC team should address the same attention to the overcrowded ED as they do to the ICUs of their hospitals.

**Table 4.** Risk Factors for Carbapenem-Resistant *Enterobacterales* Colonization on Admission in a Cohort of Patients Admitted to the Emergency Department, Hospital das Clínicas, University of São Paulo, Brazil, 2022 (n = 845)

Variable	Non-CRE Colonized on Admission (n = 817)	CRE Colonized on Admission (n = 28)	Bivariate Analysis			Multivariate Analysis		
			OR	(95% CI)	*P* Value	aOR	(95% CI)	*P* Value
Sex (female)	407 (50)	12 (43)	1.32	(.62–2.83)	.47	…	…	
Age - median (range)	58 (3–97)	63 (20–84)	…	…	.44	…	…	
Hypertension	263 (32)	10 (36)	1.17	(.53–2.57)	.69	…	…	
Heart disease	58 (7)	0 (0)	0	(0–1.91)	.25	…	…	
Lung disease	17 (2)	1 (4)	1.74	(.22–13.58)	.46	…	…	
Liver disease	30 (4)	1 (4)	0.97	(.12–7.39)	1	…	…	
Renal disease	50 (6)	1 (4)	0.57	(.08–4.27)	1	…	…	
Stroke	79 (10)	4 (14)	1.56	(.53–4.60)	.35	…	…	
Cancer	46 (6)	1 (4)	0.62	(.08–4.67)	1	…	…	
Solid organ transplantation	0 (0)	0 (0)	…	…		…	…	
Onco-hematologic disease	10 (1)	0 (0)	0	(0–13.57)	1	…	…	
Autoimmune disease	34 (4)	2 (7)	1.77	(.20–7.58)	.34	…	…	
Other comorbidities	121 (15)	6 (21)	1.57	(.51–4.10)	.29	…	…	
Exposure to the healthcare in the last year	198 (24)	11 (39)	2.03	(.93–4.39)	.07	1.95	(.89–4.24)	.09
Use of antibiotics in the last 90 d	156 (19)	10 (36)	1.04	(.99–1.08)	.048	…	…	
Category of admission								
Surgical emergency	101/783 (13)	3/26 (12)	0.85	(.16–2.87)	1	…	…	
Clinical emergency	498/783 (64)	16/26 (62)	0.85	(.40–1.83)	.70	…	…	
Neurological emergency	83/783 (11)	2/26 (8)	0.68	(.08–2.80)	1	…	…	
Pulmonary emergency	38/783 (5)	4/26 (15)	3.41	(.82–10.65)	.04	3.22	(1.06–9.81)	.04
Trauma	54/783 (7)	1/26 (4)	0.52	(.01–3.30)	1	…	…	
Acute myocardial infarction	9/783 (1)	0/26 (0)	0	(0–15.39)	1	…	…	

Data are presented as No. (%) unless otherwise indicated.

Abbreviations: aOR, adjusted odds ratio; CI, confidence interval; CRE, carbapenem-resistant *Enterobacterales*; OR, odds ratio.

## Supplementary Data

Supplementary materials are available at *Clinical Infectious Diseases* online. Consisting of data provided by the authors to benefit the reader, the posted materials are not copyedited and are the sole responsibility of the authors, so questions or comments should be addressed to the corresponding author.

## Supplementary Material

ciad263_Supplementary_Data

## Notes

***Carbapenem Resistant Enterobacterales in Emergency Department (CRE-ED) Task Force.*** Raphael B. R. Tolentino, Laina Bubach, Bianca L. Almeida, Lia M. Barreira, Priscilla C. Saihg, Roberta V. P. Yokogawa, Ana Rubia Guedes, and Thais Guimarães.

***Acknowledgments.*** The authors are thankful to Professor Heraldo Possolo de Souza, Dr Julio Cesar Alencar, and Mr Carlos Canhada, from the emergency department of Central Institute of Hospital das Clínicas da Faculdade de Medicina da Universidade de São Paulo (FMUSP); Professor Alberto José da Silva Duarte, Professor of Clinical Pathology of the FMUSP; and the infectious diseases residents. The authors also thank Fernanda C. Lessa, medical epidemiologist, acting chief of the International Control Program, Division of Healthcare Quality Promotion, Centers for Disease Control and Prevention (CDC), and Benjamin Park, senior technical advisor with the Global Fund, CDC, for revising the manuscript.

***Financial support.*** This work was supported by Fundação de Amparo à Pesquisa do Estado de São Paulo (FAPESP; grant number 2018/06016-0).

***Supplement sponsorship.*** This article appears as part of the supplement “The Evolving Challenges of Antibiotic Resistance in Low- and Middle-Income Countries: Priorities and Solutions,” sponsored by the U.S. Centers for Disease Control and Prevention, and Health Security Partners.
