# Correction to: Transmission of Carbapenem-Resistant *Enterobacterales* in an Overcrowded Emergency Department: Controlling the Spread to the Hospital

**DOI:** 10.1093/cid/ciad509

**Published:** 2023-09-01

An error appeared in a supplement article published alongside the 1 July 2023 issue of the journal (Salomão et al. “Transmission of Carbapenem-Resistant *Enterobacterales* in an Overcrowded Emergency Department: Controlling the Spread to the Hospital.” *Clin Infect Dis*; 77(Supplement_1): S46–S52. https://doi.org/10.1093/cid/ciad263). One of the members of the Carbapenem Resistant *Enterobacterales* in Emergency Department (CRE-ED) Task Force, Ana Rubia Guedes, was inadvertently left off the list of study group members include in the Notes section of the article. The full members list for the CRE-ED Task Force should read as follows:

Raphael B. R. Tolentino, Laina Bubach, Bianca L. Almeida, Lia M. Barreira, Priscilla C. Saihg, Roberta V. P. Yokogawa, Ana Rubia Guedes, Thais Guimarães.

