# Supplementary material for: Transmission of Carbapenem-Resistant Enterobacterales in an Overcrowded Emergency Department: Controlling the Spread to the Hospital
Source: Clin Infect Dis. 2023 Jul 5;77(Suppl 1):S46–52. doi: 10.1093/cid/ciad263 (PMC10321690; doi:10.1093/cid/ciad263)
Supplement: ciad263_Supplementary_Data [file ciad263_supplementary_data.docx]

Supplementary Material

**Transmission of Carbapenem-resistant *Enterobacterales* in an overcrowded emergency department: Controlling the spread to the hospital.**

Authors: Matias C. Salomão^1,2^, Maristela P. Freire^1,2^, Carolina S. Lázari^3^, Ana P. Cury^4^, Flávia Rossi^4^, Aluisio A. C. Segurado^1,5^, Silvia F. Costa^1,2,5^, Anna S. Levin^1,2,5^, Ícaro Boszczowski^1^,², on Behalf of CRE-ED Task Force

1. Department of Infectious Diseases, Faculdade de Medicina, Universidade de São Paulo, Brazil;
2. Infection Control Department, Hospital das Clínicas, Universidade de São Paulo, Brazil;
3. Divisão de Patologia Clínica – Departamento de Patologia, Laboratório de Investigação Medica (LIM03), Hospital das Clínicas, Universidade de São Paulo, Brazil;
4. Departamento de Patologia, Central Microbiology Laboratory. LIM03 Division, Hospital das Clínicas, Universidade de São Paulo, Brazil;
5. Department of Infectious Diseases, Hospital das Clínicas, Universidade de São Paulo, São Paulo, Brazil.

CRE-ED Task Force:

Raphael B. R. Tolentino^1^, Laina Bubach^1,2^, Bianca L. Almeida^2^, Lia M. Barreira^3^, Priscilla C. Saihg^3^, Roberta V. P. Yokogawa^3^, Thais Guimarães²

Contents

1. Supplementary Table 1…………………… Page 3
2. Supplementary Figure 1……………………. Page 4
3. Supplementary Figure 2……………………. Page 5

**Supplementary Table 1. Test positivity for Carbapenem Resistant *Enterobacterales* screening using RT-PCR and culture in a cohort of patients admitted to the Emergency Department, Hospital das Clínicas, University of São Paulo, 2022 (n=845)**

|  | **Positive** | **Negative** | **Positivity** |
| --- | --- | --- | --- |
| **All (845)** | | | |
| **Culture +RT-PCR** | 28 | 817 | 3.3% |
| **Culture** | 18 | 827 | 2.1% |
| **RT-PCR** | 20 | 825 | 2.4% |
| **KPC** | 17 | 828 | 2.0% |
| **NDM** | 3 | 842 | 0.4% |
| **OXA-48** | 1 | 844 | 0.1% |
| **IMP** | 0 | 845 | 0.0% |
| **VIM** | 0 | 845 | 0.0% |
| **Baseline (342)** | | | |
| **Culture +RT-PCR** | 13 | 329 | 3.8% |
| **Culture** | 9 | 333 | 2.6% |
| **RT-PCR** | 6 | 336 | 1.8% |
| **KPC** | 6 | 336 | 1.8% |
| **NDM** | 1 | 341 | 0.3% |
| **OXA-48** | 0 | 342 | 0.0% |
| **IMP** | 0 | 342 | 0.0% |
| **VIM** | 0 | 342 | 0.0% |
| **Intervention (503)** | | | |
| **Culture +RT-PCR** | 15 | 488 | 3.0% |
| **Culture** | 9 | 494 | 1.8% |
| **RT-PCR** | 14 | 489 | 2.8% |
| **KPC** | 11 | 492 | 2.2% |
| **NDM** | 2 | 501 | 0.4% |
| **OXA-48** | 1 | 502 | 0.2% |
| **IMP** | 0 | 503 | 0.0% |
| **VIM** | 0 | 503 | 0.0% |

*RT-PCR: Real time Polymerase Chain Reaction*

**Supplementary Figure 1**

Incidence of carbapenem-resistant *Klebsiella pneumoniae* infections in patients admitted to Intensive Care Units, Hospital das Clínicas University of São Paulo, São Paulo, Brazil, 2017 - 2020.


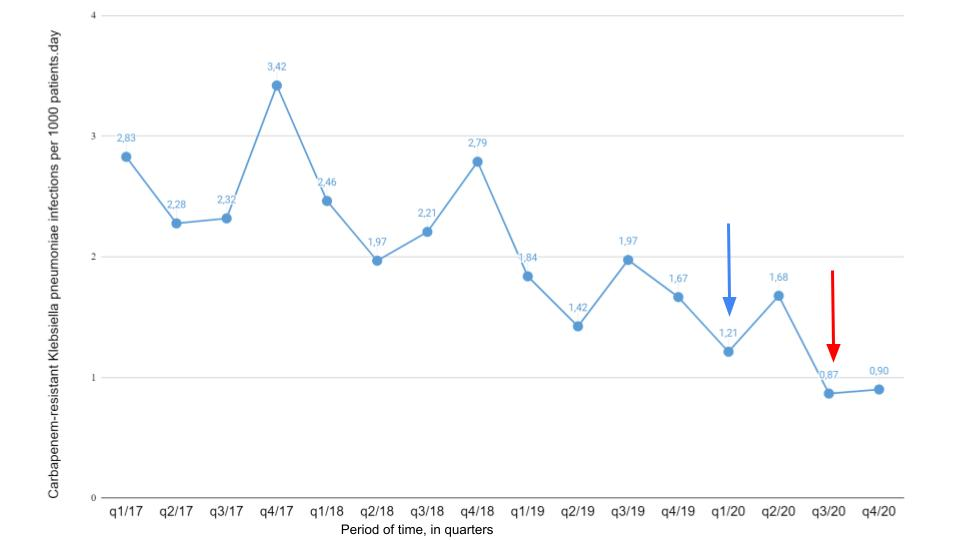


Supplemental Figure 1. Incidence of carbapenem-resistant *Klebsiella pneumoniae* infections in patients admitted to Intensive Care Units, Hospital das Clínicas University of São Paulo, São Paulo, Brazil, 2017 - 2020. Baseline is signalized in the blue arrow. Intervention is signalized in the red arrow. *q=quarter. Incidence in* carbapenem-resistant *Klebsiella pneumoniae* infections per 1000 patients.day.

**Supplementary Figure 2**

Colonization Pressure of carbapenem-resistant *Enterobacterales* in patients admitted to Intensive Care Units, Hospital das Clínicas University of São Paulo, São Paulo, Brazil, 2017 – 2020


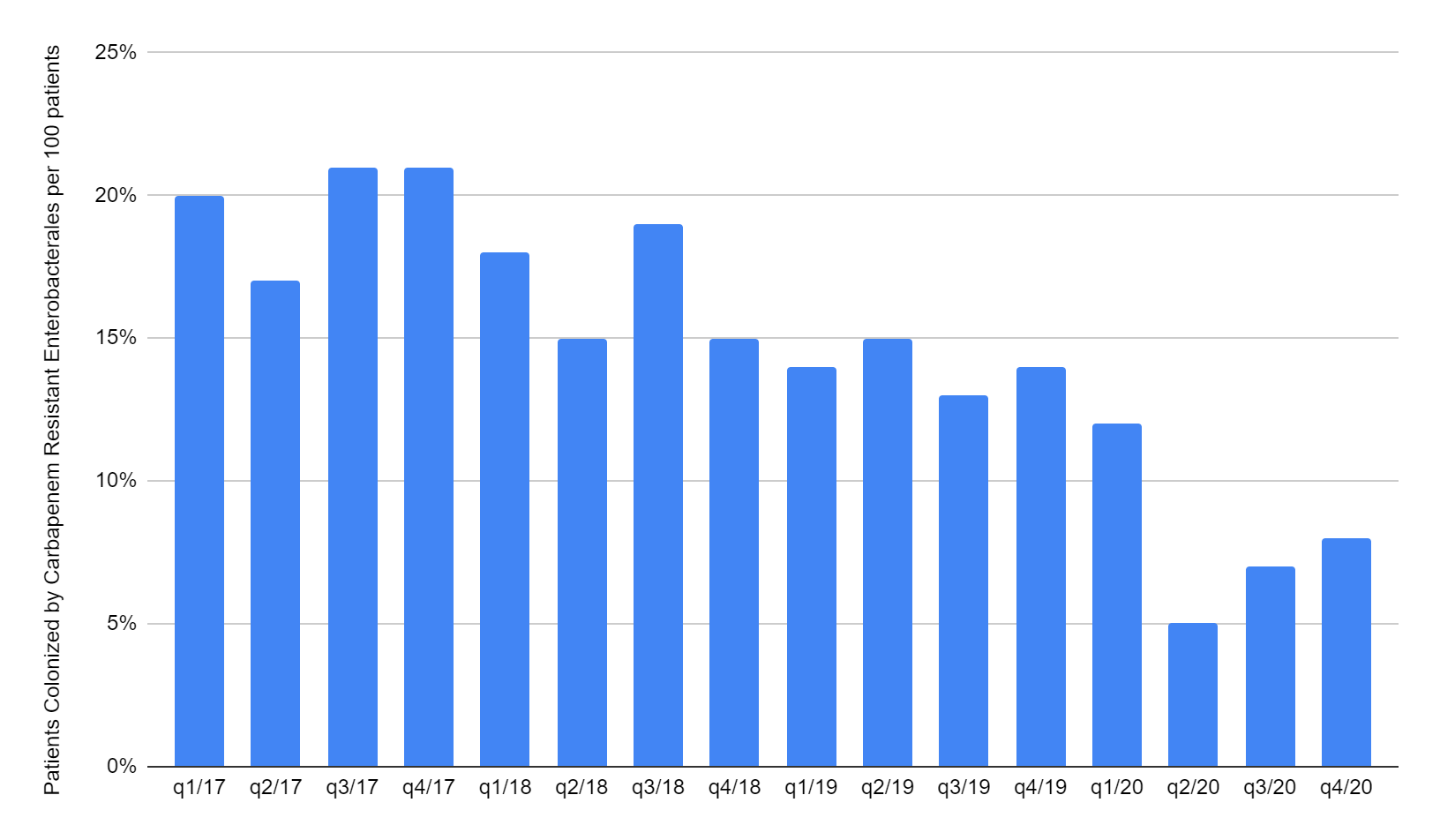


Supplemental Figure 2. Colonization Pressure of carbapenem-resistant *Enterobacterales* in patients admitted to Intensive Care Units, Hospital das Clínicas University of São Paulo, São Paulo, Brazil, 2017 - 2020. *q=quarter.*
